# Supplementary material for: Neptune’s inner moons and rings are exposed icy body interiors
Source: Sci Adv. 2026 Jul 29;12(31):eaeb1437. doi: 10.1126/sciadv.aeb1437 (PMC13418922; doi:10.1126/sciadv.aeb1437)
Supplement: Supplementary file 1 — Fig. S1 Table S1 [file sciadv.aeb1437_sm.pdf]

Science Advances
AAAS

Supplementary Materials for

Neptune's inner moons and rings are exposed icy body interiors

M. Ryleigh Davis *et al.*

Corresponding author: M. Ryleigh Davis, rdavis1@ucsd.edu

Sci. Adv. **12**, eaeb1437 (2026)
DOI: 10.1126/sciadv.aeb1437

This PDF file includes:

Fig. S1
Table S1

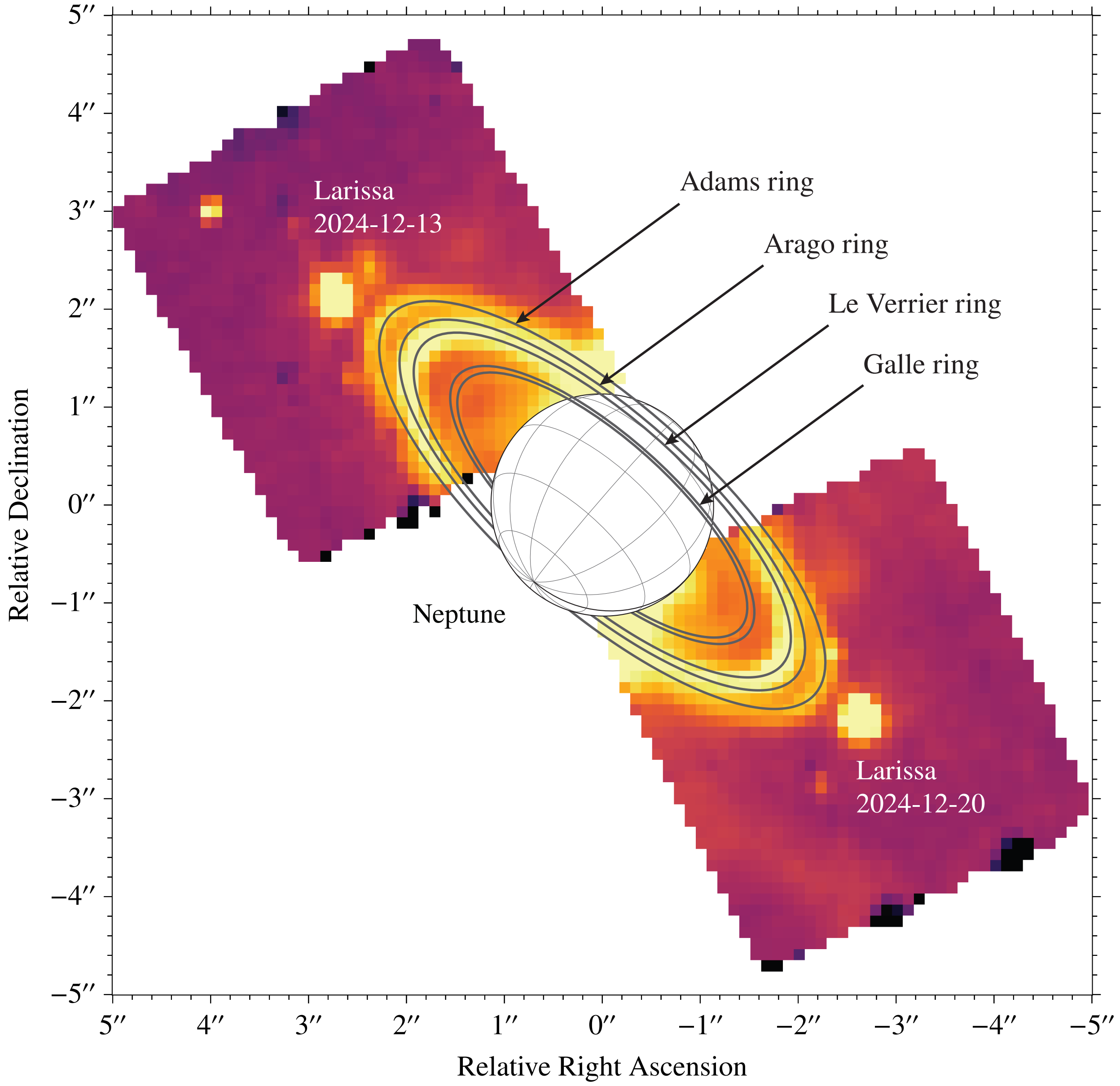

Figure S1: **Representative JWST/NIRSpec IFU images of Larissa on 2024-12-13 and 2024-12-20 showing the locations of Neptune's rings.** The IFU images were created from wavelength slices between 2.1-2.35 µm and acquired when Larissa was on opposite sides of Neptune. The bright Adams and Le Verrier rings, which are shepherded by Galatea and Despina, respectively, are easily visible in both images. The calculated ring coordinates are overlain in black.

Table S1: **Details of JWST Observations of the Inner Satellites of Neptune from program #4645.** Each individual observation is listed with the target, date in UTC, total exposure time in seconds, start and end time in UTC, and central longitude (°W) and latitude (°N/S) of the satellite in the middle of the observation. All observations were acquired using NIRSpec/IFU in PRISM mode with the clear filter.

Target	Date	Exposure Time	Start/End Time (UTC)	Obs Sub-Lon	Obs Sub-Lat
Larissa	2024-12-13	919.1 s	16:15:24/16:30:58	55°W	21°S
Larissa	2024-12-13	919.1 s	16:33:53/16:49:26	63°W	21°S
Larissa	2024-12-13	919.1 s	16:52:07/17:07:41	72°W	21°S
Proteus	2024-12-13	291.8 s	17:40:01/17:45:07	254.5°W	24.5°S
Proteus	2024-12-13	291.8 s	17:47:48/17:52:54	256°W	24.5°S
Proteus	2024-12-13	291.8 s	17:55:49/18:00:56	257.5°W	24.5°S
Proteus	2024-12-13	291.8 s	18:03:36/18:08:43	259.5°W	24.5°S
Proteus	2024-12-16	291.8 s	13:02:12/13:07:19	74.5°W	24.5°S
Proteus	2024-12-16	291.8 s	13:09:59/13:15:06	76.5°W	24.5°S
Proteus	2024-12-16	291.8 s	13:18:01/13:23:07	78°W	24.5°S
Proteus	2024-12-16	291.8 s	13:25:48/13:30:54	80°W	24.5°S
Galatea	2024-12-17	1021.2 s	15:33:55/15:51:11	226°W	20.9°S
Galatea	2024-12-17	1021.2 s	15:53:51/16:11:07	238°W	20.9°S
Galatea	2024-12-17	1021.2 s	16:13:48/16:31:03	245°W	20.9°S
Galatea	2024-12-17	1021.2 s	16:33:44/16:51:00	261°W	20.9°S
Galatea	2024-12-19	1021.2 s	08:44:32/09:01:48	226.5°W	20.9°S
Galatea	2024-12-19	1021.2 s	09:04:28/09:21:44	238°W	20.9°S
Galatea	2024-12-19	1021.2 s	09:24:24/09:41:40	250°W	20.9°S
Galatea	2024-12-19	1021.2 s	09:44:35/10:01:51	261.5°W	20.9°S
Larissa	2024-12-20	919.1 s	01:22:28/01:38:02	236°W	21°S
Larissa	2024-12-20	919.1 s	01:40:43/01:56:16	244°W	21°S
Larissa	2024-12-20	919.1 s	01:58:57/02:14:31	252°W	21°S
